# Supplementary material for: The structural basis for light acclimation in phycobilisome light harvesting systems systems in Porphyridium purpureum
Source: Commun Biol. 2023 Nov 27;6:1210. doi: 10.1038/s42003-023-05586-4 (PMC10682464; doi:10.1038/s42003-023-05586-4)
Supplement: Supplementary file 2 — Supplementary Information-New [file 42003_2023_5586_MOESM2_ESM.pdf]

The structural basis for light acclimation in phycobilisome light harvesting systems, Emma Joy Dodson, Jianfei Ma, *et al*. *Supplemental Figures and Tables*

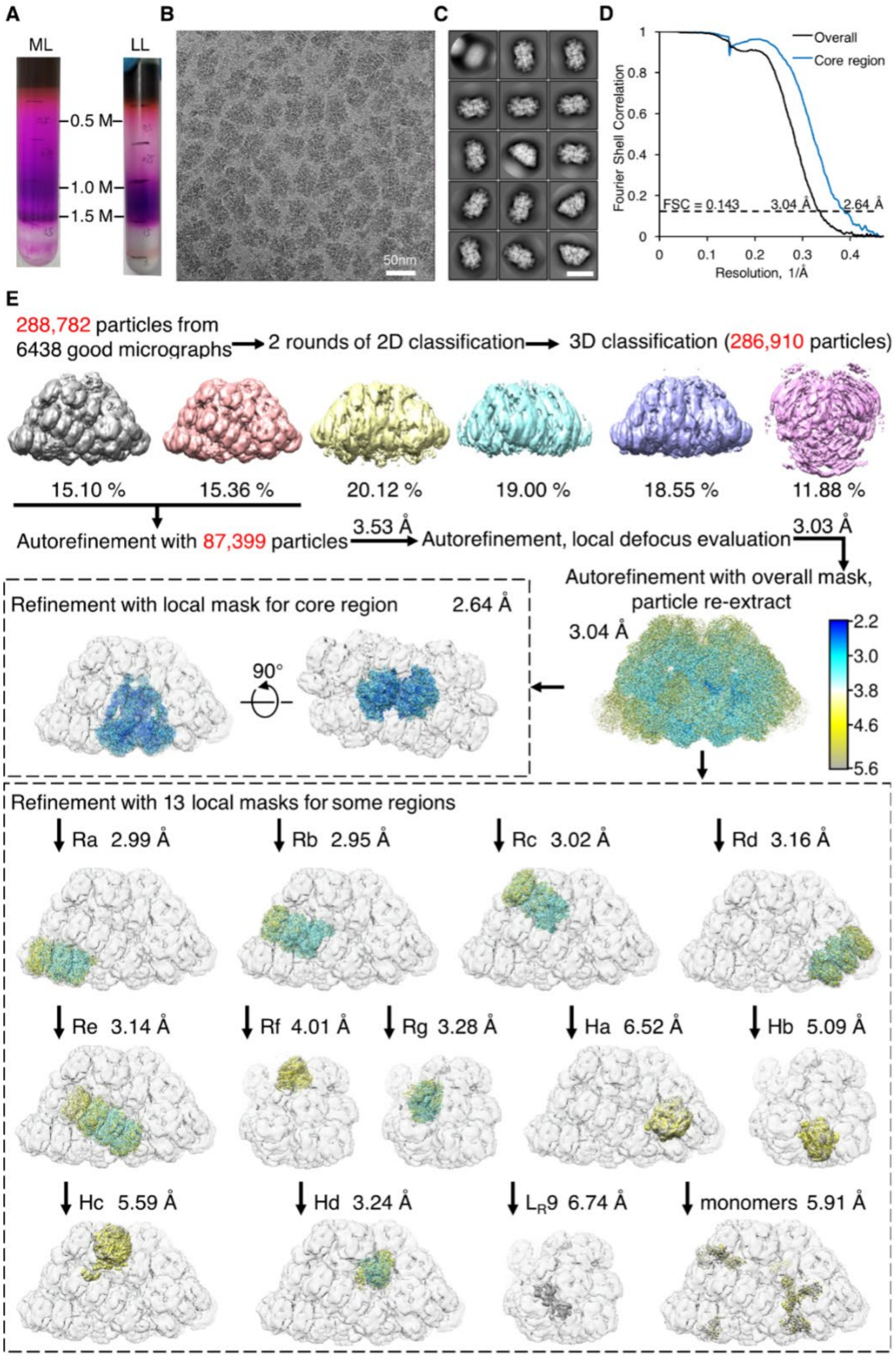

Figure S1. Cryo-EM analysis of the ML-PBS from *P. purpureum*. A. Comparison of sucrose density gradient centrifugation result between ML-PBS and LL-PBS. The violet band located at the 1.0 mol/L sucrose gradient of ML-PBS is the sample of intact PBSs used for single-particle analysis. Note that the violet band of ML-PBS is a little higher than that of LL-PBS, suggesting that the ML-PBS is lighter than LL-PBS. **B.** A representative motion-corrected electron micrograph of ML-PBSs. Scale bar, 50 nm. **C.** Typical good, reference-free 2D class averages from single-particle ML-PBS images. Scale bar, 50 nm. **D.** Gold-standard Fourier shell correlation (FSC) curves for the 3D reconstructions of the ML-PBS. Black curve, FSC curve for the overall structure; blue curve, FSC curve for the core region that was masked during refinement. **E.** The workflow for the cryo-EM data processing. For details, see 'Cryo-EM data analysis' in Methods.

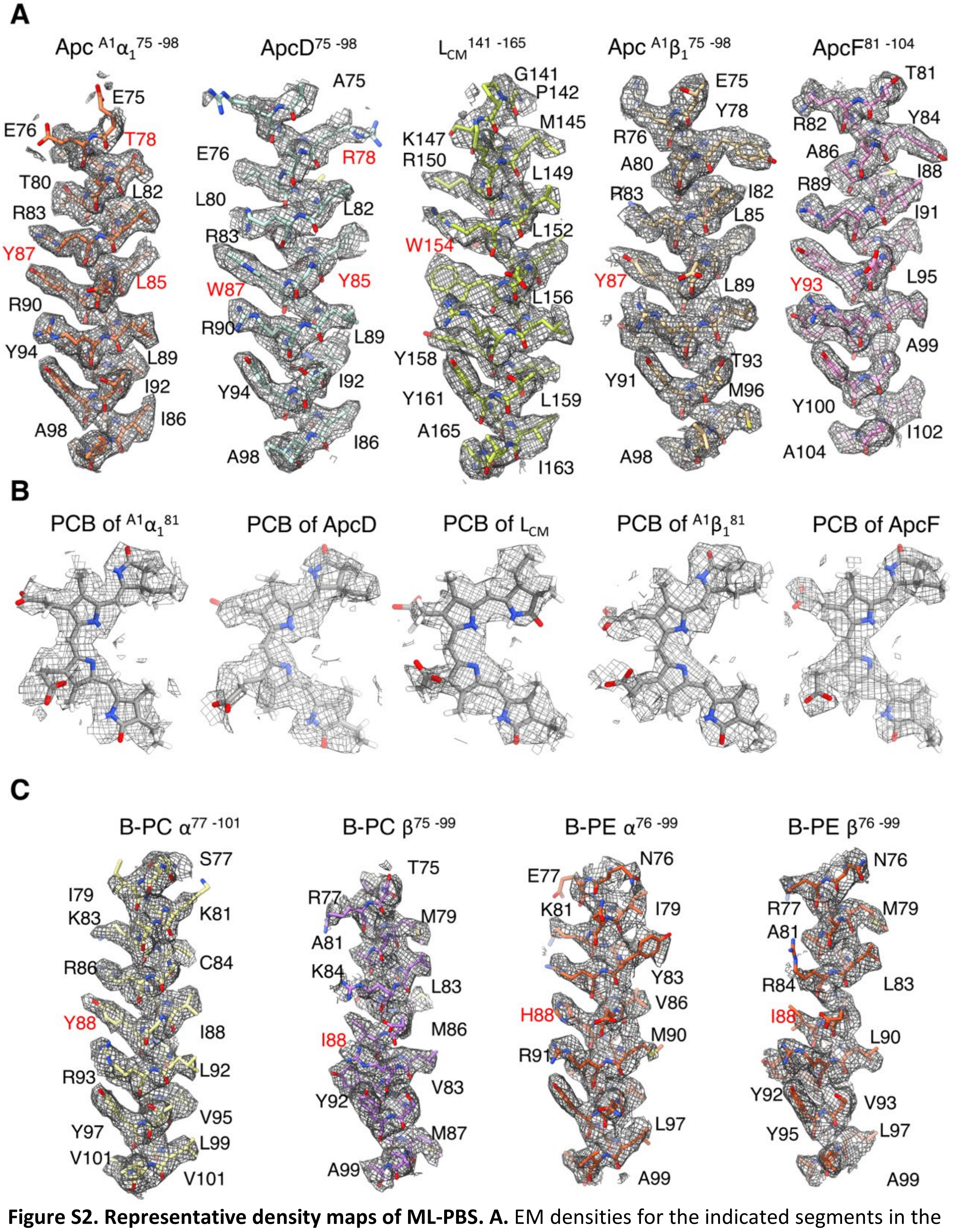

Figure S2. Representative density maps of ML-PBS. A. EM densities for the indicated segments in the core. **B.** EM densities for the indicated pigments in the core. **C.** EM densities for the indicated segments in the Rod e.

Suppl. Tab. 1: Summary of model validation for the phycobilisome components

Molecule*	MolProbity Scores	Ramachandran plot statistics (%)			RMS deviations	
		Favored	Allowed	Outliers	Bonds Length (Å)	Bonds Angles (º)
Core	2.15	96.57	3.43	0.00	0.017	2.018
Ra/Ra′	2.20	96.83	3.16	0.01	0.023	2.086
Rb/Rb′	2.26	96.65	3.25	0.10	0.021	2.001
Rc/Rc′	2.29	96.20	3.80	0.00	0.024	2.219
Rd/Rd′	2.22	96.13	3.87	0.00	0.029	2.006
Re/Re′	2.31	96.27	3.72	0.01	0.023	2.027
Rf/Rf′	2.23	97.13	2.87	0.00	0.024	1.995
Rg/Rg′	2.26	96.31	3.69	0.00	0.026	2.089
Ha/Ha′	3.00	93.17	6.79	0.04	0.025	2.283
HbM12/ HbM12′	2.46	95.37	4.63	0.00	0.023	2.091
Hc/Hc′	2.66	93.73	5.97	0.31	0.033	2.027
Hd/Hd′	2.74	94.78	4.96	0.27	0.024	2.234
Lr9/Lr9′						
M3_12/M3_12′	2.66	93.45	5.84	0.71	0.024	2.362

* Core contains all α-subunits, β-subunits in core, and L_C1/L_C1', L_{CM}/L_{CM}', $L_{RC}4/L_{RC}4'$, $L_{RC}5/L_{RC}5'$ and $L_{RC}6/L_{RC}6'$; each rod (Ra/Ra′ through Rg/Rg′) contains all α-subunits, β-subunits and linker proteins in the rod; individual hexamers (Ha/Ha′, HbM12/HbM12′, Hc/Hc′ and Hd/Hd′) contain each hexamers (Ha/Ha′, Hb/Hb′, Hc/Hc′ and Hd/Hd′) with α-subunits, β-subunits and linker proteins in the hexamers, whereas Hb/Hb′ also contains M1/M1′, M2/M2′ with β-subunits, and Hc/Hc′ contains Linker 3 (but not L_R6) as reported in revised Pp PBS model (66); Hb/Hb′ contains L_R6 as linker with the C-terminal loop domain revised as reported in revised Pp PBS model (66); L_R9/L_R9' contains L_R9/L_R9'; M3_12/M3_12′ contains each monomer (M3/M3′, M4/M4′, ⋯., M12/M12′) with α-subunits and β-subunits, and linker protein (CaRSP1, CaRSP2) as reported in revised Pp PBS model (66).

Suppl. Tab. 2: Numbers of proteins and bilins compare between LL-PBS and ML-PBS.

Subunit	Numbers in ML-PBS	Numbers in LL-PBS	Bilin									
			PCB		PEB			PUB			ML-Total	LL-Total
			Per subunit	Total	Per subunit	ML-Total	LL-Total	Per subunit	ML-Total	LL-Total		
α^{APC}	20		1	20							20	
β^{APC}	22		1	22							22	
ApcD	2		1	2							2	
ApcF	2		1	2							2	
α^{PC}	36		1	36							36	
β^{PC}	36		1	36	1	36					72	
α^{PE}	230	254			2	460	508				460	508
β^{PE}	250	274			3	750	822				750	822
L_C	2											
L_{CM}	2		1	2							2	
$L_{RC}1$	6											
$L_{RC}2$	2											
$L_{RC}3$	2											
$L_{RC}4$	2											
$L_{RC}5$	2											
$L_{RC}6$	2											
L_R1	6											
L_R2	2											
L_R3	2											
$L_R\gamma4$	6	10			3	18	30	2	12	20	30	50
$L_R\gamma5$	6				3	18		2	12		30	
L_R6	4											
$L_R\gamma7$	4				3	12		2	8		20	
$L_R\gamma8$	4				1	4		2	8		12	
L_R9	2											
Total	654	706		120		1298	1430		40	48	1458	1598

Suppl. Tab. 3: X^2 values for Lifetime results

	Type of Fit (# exp)	a1	t1 [ns]	t2 [ns]	τ- Weighted average [ns]	χ^2 (goodness of fit)	Peak Location [ns]
LL chl 1	double	1.000	0.15	0.75	0.15	3.95	1.19
LL chl 2	double	0.009	0.81	0.15	0.16	3.29	1.20
LL chl 3	double	0.988	0.15	0.80	0.16	3.19	1.20
LL pc 1	single		0.07			10.47	1.16
LL pc 2	single		0.07			13.87	1.16
LL pc 3	single		0.07			13.47	1.17
LL pe 1	single		0.04			8.89	1.13
LL pe 2	single		0.04			9.72	1.14
LL pe 3	single		0.04			9.36	1.13
ML chl 1	double	0.999	0.14	0.78	0.14	3.52	1.16
ML chl 2	double	0.002	0.74	0.13	0.13	2.65	1.19
ML chl 3	double	0.997	0.15	0.80	0.15	3.44	1.18
ML pc 1	single		0.07	0.75		15.94	1.16
ML pc 2	single		0.07			13.70	1.18
ML pc 3	single		0.07			12.89	1.17
ML pe 1	single		0.04			15.03	1.13
ML pe 2	single		0.03			12.09	1.14
ML pe 3	single		0.03			10.90	1.14

Suppl. Table 3a. Detailed TCSPC data. Data were fitted either to a single or double exponent; fit parameters displayed here are according to the following equations- single exponent: $I(t) = exp\left(-\frac{t}{t1}\right), \tau = t1$, double exponent: $I(t) = a1 * exp\left(-\frac{t}{t1}\right) + (1-a1) * exp\left(-\frac{t}{t2}\right)$. Weighted average calculation for lifetimes: $\tau = a1 * t1 + (1-a1) * t2$.

	Average τ [ns]	Std of average τ (sample)	Average peak location [ns]	Std of average peak location (sample)
LL chl	0.16	0.01	1.20	0.01
LL pc	0.07	0.00	1.16	0.01
LL pe	0.04	0.00	1.13	0.01
ML chl	0.14	0.01	1.18	0.02
ML pc	0.07	0.00	1.17	0.01
ML pe	0.03	0.00	1.14	0.01

Suppl. Table 3b. Average lifetimes and peak locations for TCSPC data. (n=3 for each type of measurement).

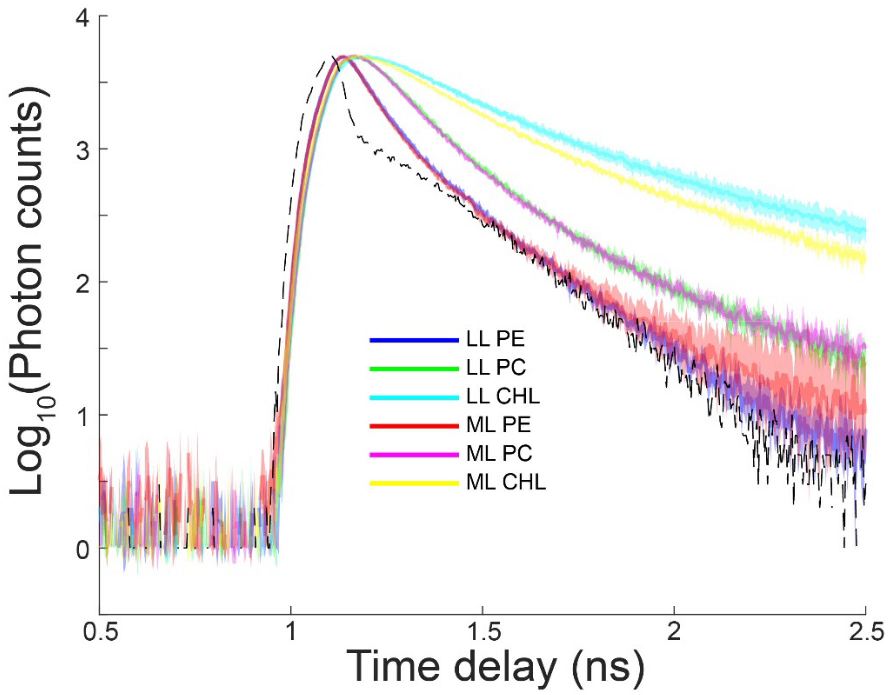

Figure S3. Lifetime graph with Y axis drawn in logarithmic scale. Fluorescence lifetime was studied by TCSPC. Excitation was at 495 nm and the data was collected in three detection windows: Preferential PE emission (PE 515–575 nm), Preferential PC emission (PC 650-670 nm) or preferential chlorophyll+APC emission (CHL 675–750 nm). The shaded areas represent standard deviation. The dashed black line shows the Instrument Response Function (IRF).
