# Supplementary material for: The structural basis for light acclimation in phycobilisome light harvesting systems systems in Porphyridium purpureum
Source: Commun Biol. 2023 Nov 27;6:1210. doi: 10.1038/s42003-023-05586-4 (PMC10682464; doi:10.1038/s42003-023-05586-4)
Supplement: Supplementary file 5 — Reporting summary [file 42003_2023_5586_MOESM5_ESM.pdf]

nature portfolio

Corresponding author(s): Nir Keren

Last updated by author(s): 09-19-2023

Reporting Summary

Nature Portfolio wishes to improve the reproducibility of the work that we publish. This form provides structure for consistency and transparency in reporting. For further information on Nature Portfolio policies, see our Editorial Policies and the Editorial Policy Checklist.

Statistics

For all statistical analyses, confirm that the following items are present in the figure legend, table legend, main text, or Methods section.

n/a	Confirmed	
☐	☒	The exact sample size (n) for each experimental group/condition, given as a discrete number and unit of measurement
☐	☒	A statement on whether measurements were taken from distinct samples or whether the same sample was measured repeatedly
☒	☐	The statistical test(s) used AND whether they are one- or two-sided<br>*Only common tests should be described solely by name; describe more complex techniques in the Methods section.*
☒	☐	A description of all covariates tested
☒	☐	A description of any assumptions or corrections, such as tests of normality and adjustment for multiple comparisons
☒	☐	A full description of the statistical parameters including central tendency (e.g. means) or other basic estimates (e.g. regression coefficient) AND variation (e.g. standard deviation) or associated estimates of uncertainty (e.g. confidence intervals)
☒	☐	For null hypothesis testing, the test statistic (e.g. F, t, r) with confidence intervals, effect sizes, degrees of freedom and P value noted<br>*Give P values as exact values whenever suitable.*
☒	☐	For Bayesian analysis, information on the choice of priors and Markov chain Monte Carlo settings
☒	☐	For hierarchical and complex designs, identification of the appropriate level for tests and full reporting of outcomes
☒	☐	Estimates of effect sizes (e.g. Cohen's d, Pearson's r), indicating how they were calculated

Our web collection on statistics for biologists contains articles on many of the points above.

Software and code

Policy information about availability of computer code

Data collection	Micrograph screening, particle picking and normalization were performed by Relion 3.0 beta (Scheres, 2012; Scheres, 2012). 6,438 micrographs were selected for further processing.
Data analysis	The contrast transfer function parameters of each micrograph were estimated by CTFFIND4 (Mindell, 2003) and all the 2D, 3D classification and refinement, CTF refinement were performed with Relion3.0 beta.

For manuscripts utilizing custom algorithms or software that are central to the research but not yet described in published literature, software must be made available to editors and reviewers. We strongly encourage code deposition in a community repository (e.g. GitHub). See the Nature Portfolio guidelines for submitting code & software for further information.

Data

Policy information about availability of data

All manuscripts must include a data availability statement. This statement should provide the following information, where applicable:

- Accession codes, unique identifiers, or web links for publicly available datasets
- A description of any restrictions on data availability
- For clinical datasets or third party data, please ensure that the statement adheres to our policy

Both low light and medium light models can be found in the Protein Data Bank rcsb.org (Berman H.M., 2000) (PDB 6KGX and PDB 7EZX, respectively).

Human research participants

Policy information about studies involving human research participants and Sex and Gender in Research.

Reporting on sex and gender	Not Relevant
Population characteristics	Not Relvenat
Recruitment	Not Relevant
Ethics oversight	Not Relevant

Note that full information on the approval of the study protocol must also be provided in the manuscript.

Field-specific reporting

Please select the one below that is the best fit for your research. If you are not sure, read the appropriate sections before making your selection.

☐ Life sciences ☐ Behavioural & social sciences ☐ Ecological, evolutionary & environmental sciences

For a reference copy of the document with all sections, see nature.com/documents/nr-reporting-summary-flat.pdf

Life sciences study design

All studies must disclose on these points even when the disclosure is negative.

Sample size	Detailed in figure legends
Data exclusions	Data was no Excluded
Replication	Detailed in figure legends
Randomization	Not required in this study
Blinding	Not required in this study

Behavioural & social sciences study design

All studies must disclose on these points even when the disclosure is negative.

Study description	Not relevant
Research sample	Not relevant
Sampling strategy	Not relevant
Data collection	Not relevant
Timing	Not relevant
Data exclusions	Not relevant
Non-participation	Not relevant
Randomization	Not relevant

Ecological, evolutionary & environmental sciences study design

All studies must disclose on these points even when the disclosure is negative.

Study description	Not relevant
Research sample	Not relevant
Sampling strategy	Not relevant
Data collection	Not relevant
Timing and spatial scale	Not relevant
Data exclusions	Not relevant
Reproducibility	Not relevant
Randomization	Not relevant
Blinding	Not relevant

Did the study involve field work? ☐ Yes ☒ No

Reporting for specific materials, systems and methods

We require information from authors about some types of materials, experimental systems and methods used in many studies. Here, indicate whether each material, system or method listed is relevant to your study. If you are not sure if a list item applies to your research, read the appropriate section before selecting a response.

Materials & experimental systems

n/a	Involved in the study
☒	☐ Antibodies
☒	☐ Eukaryotic cell lines
☒	☐ Palaeontology and archaeology
☒	☐ Animals and other organisms
☒	☐ Clinical data
☒	☐ Dual use research of concern

Methods

n/a	Involved in the study
☒	☐ ChIP-seq
☒	☐ Flow cytometry
☒	☐ MRI-based neuroimaging
